# Association between tooth loss rate and risk of mild cognitive impairment in older adults: a population-based longitudinal study

**DOI:** 10.18632/aging.203504

**Published:** 2021-09-07

**Authors:** Shuyu Xu, Xi Huang, Yin Gong, Jiangwei Sun

**Affiliations:** 1 Department of Implantology, School and Hospital of Stomatology, Shanghai Engineering Research Center of Tooth Restoration and Regeneration, Tongji University, Shanghai, China; 2 Department of Stomatology, First Affiliated Hospital of Soochow University, Jiangsu Province, China; 3 Institute of Environmental Medicine, Karolinska Institute, Stockholm, Sweden

**Keywords:** tooth loss, mild cognitive impairment, longitudinal study, elderly, China

## Abstract

Mild cognitive impairment (MCI) is a symptomatic predementia phase of the trajectory of cognitive decline, and its prevalence increases with age. Although the relationship between oral health and MCI have been explored previously, it is uncertain whether individuals with different tooth loss rates have altered MCI risks. We hereby conducted a longitudinal study by using data from the Chinese Longitudinal Healthy Longevity Survey to investigate the association. Tooth loss rate was defined as the difference of teeth between two interview waves divided by years of interval; participants were then grouped into four categories: stable, no tooth loss; mild, 0-1 tooth loss; middle, 1-2 tooth loss; and severe, more than 2 tooth loss per year. Cognitive function was assessed by the Chinese version of Mini-Mental State Examination. We used the generalized estimating equation model to estimate the odds ratio (OR) and the 95% confidence intervals (CIs) and applied the restricted cubic spline function to explore the dose-response association. Among 11,862 participants, 3,966 developed MCI in a median follow-up time of 5.93 years. Higher tooth loss rate was associated with an increased risk of MCI in elderly subjects. Compared with subjects with stable tooth, the corresponding ORs (95% CIs) were 0.94 (0.85-1.03), 1.16 (1.04-1.29) and 1.28 (1.17-1.40) for subjects with the mild, middle and severe rate of tooth loss. A nonlinear dose-response relationship was detected (*P*_non-linearity_ = 0.0165). Similar results were observed in the subgroup analyses stratified by sex, age at baseline, and number of teeth at baseline. The positive association was only observed among denture nonwearers (OR _middle vs stable_: 1.19; 1.06-1.35; OR _severe vs stable_: 1.35; 1.22-1.50), but not among denture wearers. In conclusion, among elderly population in China, higher rate of tooth loss may be associated with an increased risk of MCI, while denture wearers may be less likely to develop MCI.

## INTRODUCTION

Aging, a natural process of life, is often accompanied by various disorders or diseases including cognitive impairment [1]. Cognitive impairment is characterized by the gradual loss of one’s ability in learning, remembering, concentrating, and making decisions [2]. Mild cognitive impairment (MCI) is the clinical stage between the expected cognitive decline of normal aging and dementia [2]. Its incidence rate widely ranges by age [3]: 22.5 per 1,000 person-years for aged 75-79, 40.9 for aged 80-84, and 60.1 for aged over 85 years [3]. In China, the incidence rate of MCI was estimated to be 70.57 per 1,000 person-years for aged over 60 years, [4] with a prevalence of 15·5%, representing 38·77 million people [5]. With huge impacts on the quality of life for patients, the global economic burden of dementia is estimated to be more than 800 billion USD [6].

The strongest risk factors of MCI included old age and having a specific form of a gene known as APOE e4, which is also linked to Alzheimer's disease [7–9]. Epidemiological studies have also identified other medical conditions and lifestyles as risk factors for MCI, including high blood pressure, diabetes mellitus, depression, low education level, less frequent participation in stimulating activities mentally or socially, smoking, low vegetable and high saturated fat consumption [8–10].

Oral diseases are one age-related health problem that is common among elderly adults [11]. The need for dental treatment however has been unmet among elderly people, especially in the developing countries [11, 12]. Previous studies have suggested that oral health is associated with cognitive function, but the results of periodontal disease were conflicting [13]. Recent prospective evidence indicated that less teeth at baseline may be associated with cognitive decline in elderly people [14], this hypothesis was further supported by the evidence from a meta-analysis, which showed that tooth loss was related to an increased risk of MCI and dementia [13]. Evidence from longitudinal studies revealed that denture wearing, through increasing nutritional intake and encouraging social participation, was associated with lower risk of MCI and other health-related outcomes such as cardiovascular and respiratory disease mortality [15, 16]. However, another longitudinal study, conducted in participants aged 70-79 years in US, did not report a significant association between tooth loss and cognitive decline [17].

As previous studies varied in study quality and result, the relationship between tooth loss and MCI risk in elderly adults remains inconclusive. Besides, previous studies usually use number of teeth in a specific time point (e.g., baseline) as the exposure to reflect the oral health status [14, 18], which cannot track the dynamic change of teeth with aging. Number of tooth loss per year may be a more suitable indicator to reflect its dynamic change.

Therefore, to explore the effect of change in teeth number on MCI risk, we conducted a longitudinal study to explore their associations in elderly Chinese population. The dose-response association between them was also investigated in the present study.

## RESULTS

### Baseline characteristics of study population

**Figure 1:**
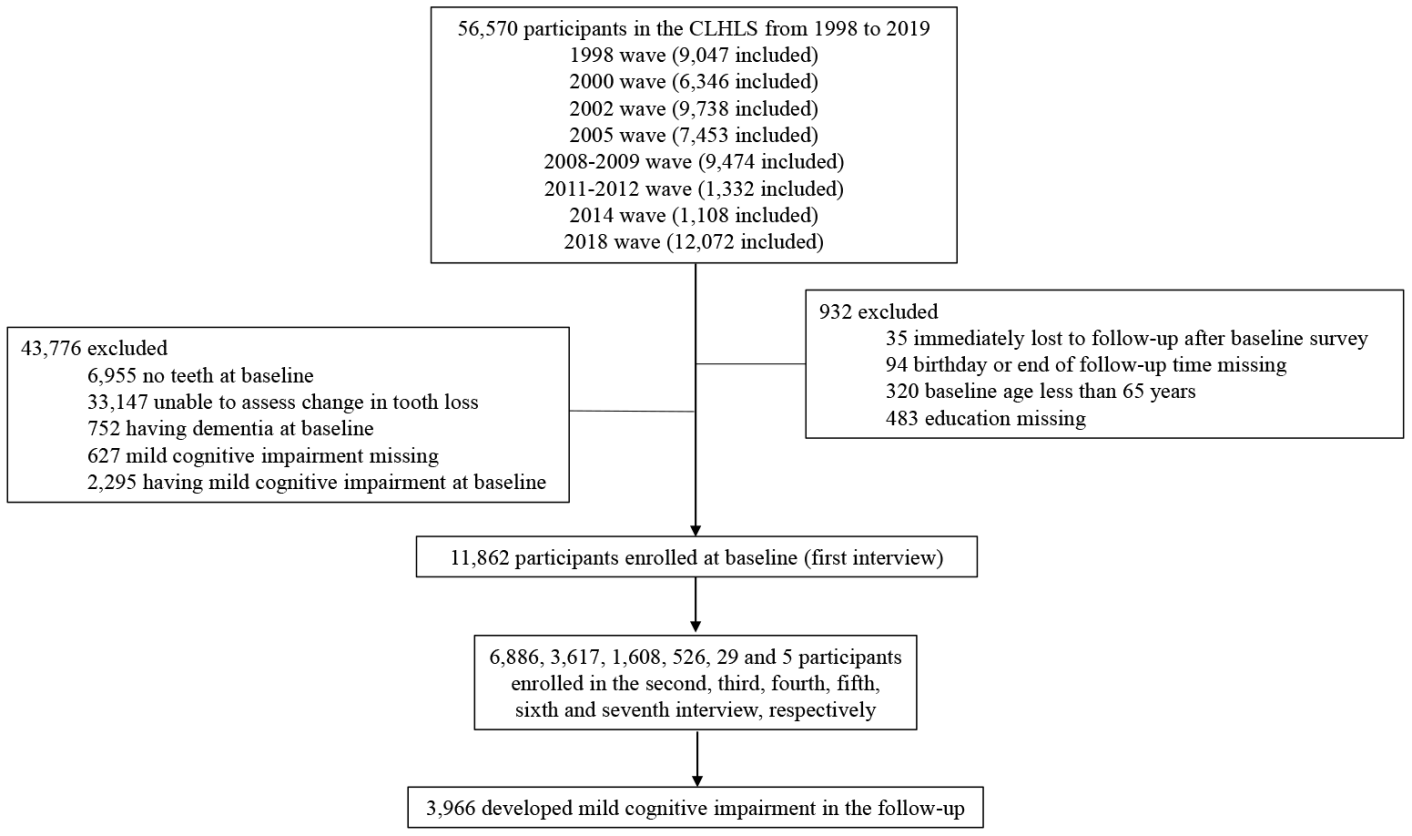
Flow chart of selecting study population from the Chinese longitudinal healthy longevity survey during 1998-2019.

Among 11,862 participants [age at baseline: 81.41 years; male: 6,047 (50.98%)], 3,966 developed MCI in a median follow-up time of 5.93 (interquartile range: 3.67-9.62) years (Table 1 and Figure 1). A total of 4,705, 2,016, 1,687, and 3,454 subjects were classified into groups of stable, mild, middle, and severe rate of tooth loss, respectively. Compared with participants with stable status, participants with severe rate of tooth loss were more likely to be younger, to be farmer or manual workers, to live in rural area, to have more teeth and higher physical performance score and food diversity score at baseline; but less likely to be married, ever or current exerciser, and to wear denture at baseline (Table 1).

**Table 1:** Baseline characteristics in elderly adults, a longitudinal study in China, 1998 to 2019.

**Variables**	**Whole population (n=11,862)**	**Tooth lose rate, per year**				**P value**
		**Stable (n=4,705)**	**Mild (n=2,016)**	**Middle (n=1,687)**	**Severe (n=3,454)**	
Age at baseline, years						
Mean ± SD	81.41 ± 10.47	81.39 ± 10.38	83.21 ± 10.87	81.55 ± 10.68	80.31 ± 10.11	<0.0001
Median (IQR)	81.48 (72.10-89.08)	81.59 (72.36-88.84)	83.60 (74.00-91.13)	81.81 (71.79-89.89)	80.54 (71.19-87.39)	
<80 years	4942 (41.66)	1914 (40.68)	718 (35.62)	707 (41.91)	1603 (46.41)	
80-89 years	4225 (35.62)	1749 (37.17)	716 (35.52)	568 (33.67)	1192 (34.51)	
≥90 years	2695 (22.72)	1042 (22.15)	582 (28.87)	412 (24.42)	659 (19.08)	
Male, n(%)	6047 (50.98)	2433 (51.71)	944 (46.83)	851 (50.44)	1819 (52.66)	0.2888
Ethic, Han, n(%)	11183 (94.28)	4395 (93.41)	1892 (93.85)	1603 (95.02)	3293 (95.34)	<0.0001
Marriage status, married, n(%)	6405 (54.00)	2575 (54.73)	1176 (58.33)	923 (54.71)	1731 (50.12)	<0.0001
Ever or current smoker, n(%)	4459 (37.64)	1821 (38.73)	721 (35.80)	586 (34.80)	1331 (38.60)	0.6258
Ever or current drinker, n(%)	4092 (34.55)	1703 (36.24)	660 (32.82)	569 (33.79)	1160 (33.63)	0.0173
Ever or current exerciser, n(%)	4720 (39.89)	1919 (40.92)	794 (39.44)	693 (41.15)	1314 (38.14)	0.0285
High ADL score, n(%)	10737 (90.52)	4261 (90.56)	1784 (88.49)	1531 (90.75)	3161 (91.52)	0.0785
No. of teeth at baseline, ≥ 17, n(%)	4397 (37.07)	1268 (26.95)	463 (22.97)	533 (31.59)	2133 (61.75)	<0.0001
Denture wearing, yes, n(%)	2294 (19.34)	952 (20.23)	379 (18.80)	353 (20.92)	610 (17.66)	0.0157
Residence, n(%)						0.0002
City	2860 (24.11)	1172 (24.91)	472 (23.41)	429 (25.43)	787 (22.79)	
Town	3396 (28.63)	1385 (29.44)	621 (30.80)	460 (27.27)	930 (26.93)	
Rural area	5606 (47.26)	2148 (45.65)	923 (45.78)	798 (47.30)	1737 (50.29)	
Education, n(%)						0.4667
Illiterate	6649 (56.05)	2582 (54.88)	1238 (61.41)	907 (53.76)	1922 (55.65)	
Primary school	3927 (33.11)	1572 (33.41)	597 (29.61)	565 (33.49)	1193 (34.54)	
Middle school or above	1286 (10.84)	551 (11.71)	181 (8.98)	215 (12.74)	339 (9.81)	
Occupation, n(%)						0.0033
Farmer or manual	6495 (54.75)	2475 (52.60)	1135 (56.30)	925 (54.83)	1960 (56.75)	
Clerical	3197 (26.95)	1336 (28.40)	546 (27.08)	425 (25.19)	890 (25.77)	
Professional	1122 (9.46)	487 (10.35)	147 (7.29)	179 (10.61)	309 (8.95)	
Others	1048 (8.83)	407 (8.65)	188 (9.33)	158 (9.37)	295 (8.54)	
Physical performance score, n(%)						0.0283
5	8659 (73.00)	3436 (73.03)	1411 (69.99)	1197 (70.95)	2615 (75.71)	
2.5-4.5	3047 (25.69)	1212 (25.76)	576 (28.57)	465 (27.56)	794 (22.99)	
0-2.5	156 (1.32)	57 (1.21)	29 (1.44)	25 (1.48)	45 (1.30)	
Food diversity score, n(%)						0.0002
6-8	5995 (50.54)	2350 (49.95)	947 (46.97)	822 (48.73)	1876 (54.31)	
4-5	4032 (33.99)	1614 (34.30)	727 (36.06)	592 (35.09)	1099 (31.82)	
0-3	1835 (15.47)	741 (15.75)	342 (16.96)	273 (16.18)	479 (13.87)	
Social activity score, n(%)						0.7672
5-8	3213 (27.09)	1301 (27.65)	472 (23.41)	474 (28.10)	966 (27.97)	
3-4	6274 (52.89)	2556 (54.33)	1039 (51.54)	875 (51.87)	1804 (52.23)	
0-2	2375 (20.02)	848 (18.02)	505 (25.05)	338 (20.04)	684 (19.80)	
Chronic disease score, n(%)						0.4043
0	7006 (59.06)	2781 (59.11)	1203 (59.67)	978 (57.97)	2044 (59.18)	
1-2	4291 (36.17)	1725 (36.66)	707 (35.07)	628 (37.23)	1231 (35.64)	
≥3	565 (4.76)	199 (4.23)	106 (5.26)	81 (4.80)	179 (5.18)	

Abbreviations: ADL, activities of daily living; IQR, interquartile range; SD, standard deviation.

### Association between tooth loss rate and risk of MCI

**Figure 2:**
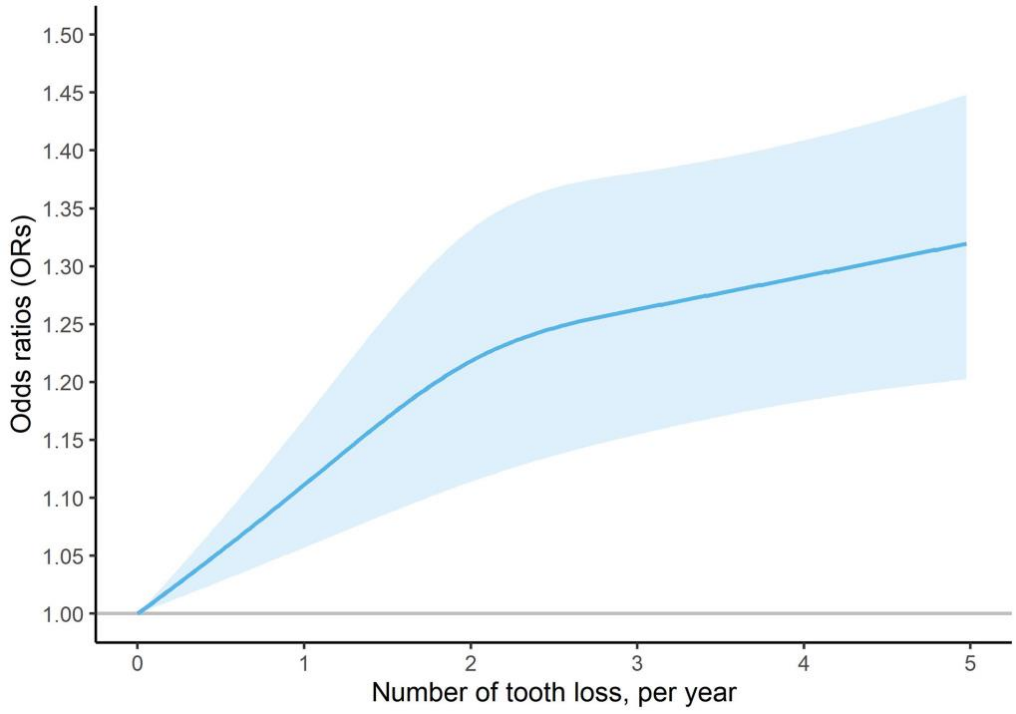
Dose-response association of number of tooth loss (per year) and risk of mild cognitive impairment in the whole population.

Results from model 1 to model 4 was robust. We found that higher tooth loss rate was associated with an increased risk of MCI in elderly participants. Compared with subjects with stable teeth, the corresponding ORs were 0.94 (95%CI: 0.85-1.03), 1.16 (95%CI: 1.04-1.29) and 1.28 (95%CI: 1.17-1.40) for subjects with the mild, middle and severe rate of tooth loss in model 4 (Table 2). As shown in Figure 2, there was a nonlinear dose-response relationship between number of tooth loss (per year) and risk of MCI in the whole population (*P*_non-linearity_ = 0.0165). The risk of MCI sharply increased in the initial stage but gradually tended to be flat with the increasing of number of tooth loss.

**Table 2:** Association of tooth lose rate with mild cognitive impairment in elderly adults, a longitudinal study in China, 1998 to 2019.

**Population**	**Tooth lose rate**	**Odds ratio (95% confidence intervals)**			
		**Model 1**	**Model 2**	**Model 3**	**Model 4**
Whole population					
	Stable	1 (Ref.)	1 (Ref.)	1 (Ref.)	1 (Ref.)
	Mild	0.94 (0.86-1.04)	0.94 (0.86-1.03)	0.95 (0.86-1.05)	0.94 (0.85-1.03)
	Middle	1.11 (1.00-1.23)	1.11 (1.00-1.23)	1.16 (1.04-1.29)	1.16 (1.04-1.29)
	Severe	1.12 (1.03-1.22)	1.14 (1.04-1.24)	1.22 (1.12-1.33)	1.28 (1.17-1.40)
Age at baseline, 65-79 years					
	Stable	1 (Ref.)	1 (Ref.)	1 (Ref.)	1 (Ref.)
	Mild	0.85 (0.70-1.03)	0.84 (0.69-1.01)	0.83 (0.68-1.01)	0.81 (0.67-0.99)
	Middle	1.13 (0.93-1.37)	1.12 (0.93-1.36)	1.15 (0.94-1.40)	1.14 (0.93-1.39)
	Severe	1.23 (1.06-1.44)	1.26 (1.08-1.47)	1.29 (1.10-1.52)	1.33 (1.13-1.56)
Age at baseline, 80-89 years					
	Stable	1 (Ref.)	1 (Ref.)	1 (Ref.)	1 (Ref.)
	Mild	0.98 (0.85-1.12)	0.98 (0.85-1.13)	0.99 (0.86-1.15)	0.97 (0.84-1.12)
	Middle	1.04 (0.89-1.21)	1.04 (0.89-1.22)	1.12 (0.95-1.31)	1.12 (0.95-1.32)
	Severe	1.15 (1.01-1.31)	1.17 (1.02-1.33)	1.26 (1.10-1.44)	1.33 (1.15-1.53)
Age at baseline, ≥ 90 years					
	Stable	1 (Ref.)	1 (Ref.)	1 (Ref.)	1 (Ref.)
	Mild	1.00 (0.84-1.19)	1.00 (0.84-1.18)	1.02 (0.85-1.22)	1.01 (0.84-1.21)
	Middle	1.24 (1.02-1.51)	1.23 (1.01-1.50)	1.28 (1.04-1.57)	1.28 (1.04-1.57)
	Severe	1.05 (0.89-1.24)	1.07 (0.90-1.27)	1.17 (0.98-1.40)	1.23 (1.03-1.48)
Male					
	Stable	1 (Ref.)	1 (Ref.)	1 (Ref.)	1 (Ref.)
	Mild	0.95 (0.83-1.10)	0.95 (0.82-1.09)	0.98 (0.85-1.14)	0.97 (0.83-1.12)
	Middle	1.25 (1.08-1.45)	1.25 (1.07-1.45)	1.28 (1.10-1.50)	1.28 (1.10-1.49)
	Severe	1.07 (0.94-1.21)	1.09 (0.96-1.23)	1.17 (1.02-1.33)	1.20 (1.04-1.37)
Female					
	Stable	1 (Ref.)	1 (Ref.)	1 (Ref.)	1 (Ref.)
	Mild	0.94 (0.82-1.06)	0.93 (0.82-1.06)	0.94 (0.82-1.07)	0.92 (0.81-1.05)
	Middle	1.01 (0.87-1.16)	1.00 (0.87-1.16)	1.06 (0.92-1.23)	1.06 (0.92-1.23)
	Severe	1.17 (1.04-1.31)	1.19 (1.06-1.34)	1.28 (1.13-1.45)	1.37 (1.20-1.55)

Model 1: adjusted for age, sex, and enrollment year; Model 2: model 1 + further adjusted for province, residence, ethic, marriage status, occupation, education; Model 3: model 2 + further adjusted for ADL score, physical performance score, food diversity score, social activity score, and chronic disease score; Model 4: model 3 + further adjusted for No. of teeth at baseline and denture wearing at baseline.

**Figure 3:**
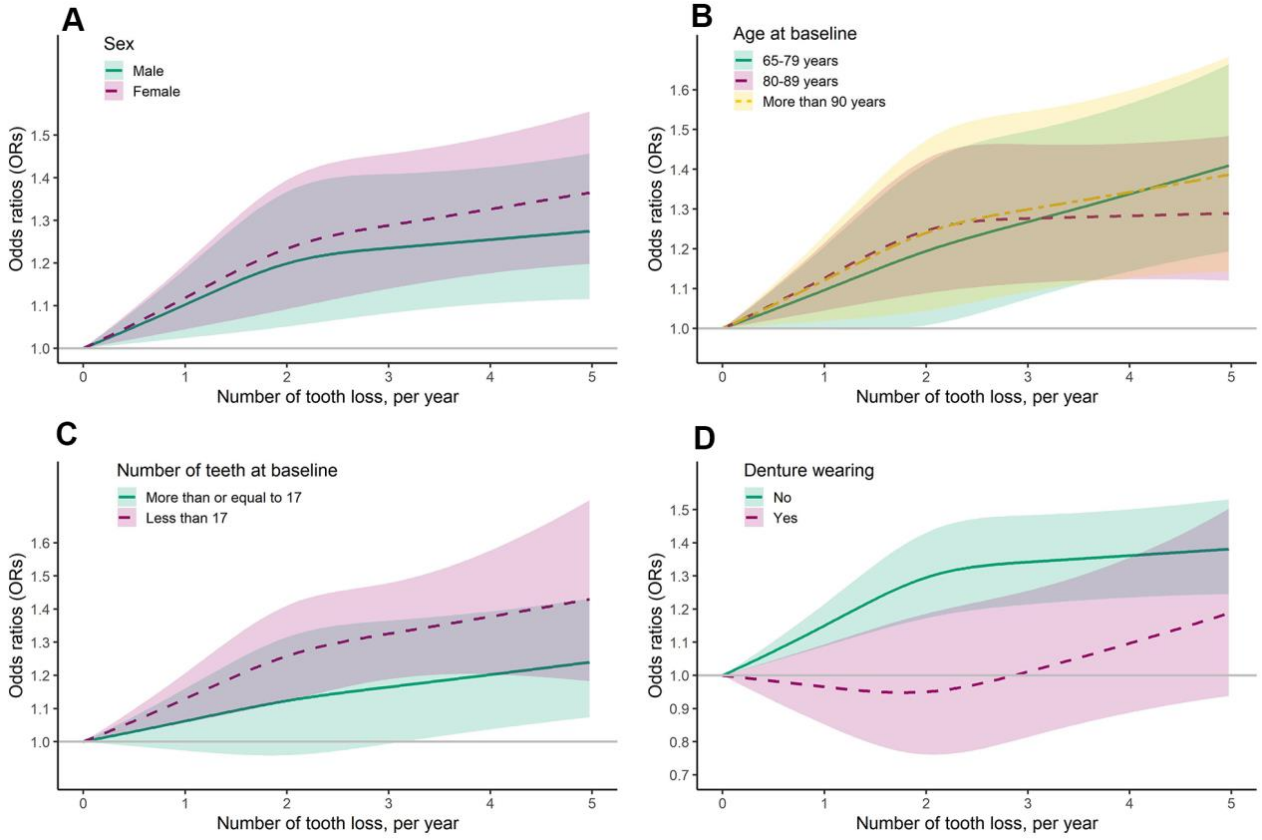
Dose-response association of number of tooth loss (per year) and risk of mild cognitive impairment, stratified by sex (**A**), age (**B**), number of teeth (**C**), and denture wearing (**D**) at baseline.

Similar results were also observed in the subgroup analyses based on sex and age at baseline. For severe rate of tooth loss, there was a 20% increased risk of MCI (1.04-1.37) for men, while 37% (1.20-1.55) for women. The corresponding ORs were 1.33 (1.13-1.56), 1.33 (1.15-1.53) and 1.23 (1.03-1.48) for participants with 65-79 years, 80-89 years, and ≥ 90 years at baseline (Table 2 and Figure 3).

In the subgroup analysis by number of teeth at baseline, we found that severe rate of tooth loss was associated with increased risk of MCI, and the risk was higher for subjects with number of teeth less than 17 (1.34; 1.18-1.51) than for subjects with number of teeth more than 17 at baseline (1.21; 1.05-1.41) (Table 3). Similar result was also observed in the dose-response analysis (Figure 3).

**Table 3:** Association of tooth lose rate with mild cognitive impairment in elderly adults, stratified by number of teeth and denture wearing at baseline.

**Population**	**Tooth lose rate**	**Odds ratio (95% confidence intervals)**			
		**Model 1**	**Model 2**	**Model 3**	**Model 4**
No. of teeth at baseline, ≥ 17					
	Stable	1 (Ref.)	1 (Ref.)	1 (Ref.)	1 (Ref.)
	Mild	0.86 (0.70-1.06)	0.86 (0.69-1.06)	0.84 (0.67-1.05)	0.83 (0.67-1.04)
	Middle	1.16 (0.95-1.42)	1.16 (0.94-1.42)	1.16 (0.94-1.44)	1.15 (0.93-1.42)
	Severe	1.17 (1.01-1.34)	1.19 (1.03-1.37)	1.23 (1.06-1.42)	1.21 (1.05-1.41)
No. of teeth at baseline, < 17					
	Stable	1 (Ref.)	1 (Ref.)	1 (Ref.)	1 (Ref.)
	Mild	0.96 (0.86-1.06)	0.95 (0.86-1.06)	0.98 (0.87-1.09)	0.97 (0.87-1.08)
	Middle	1.10 (0.98-1.24)	1.10 (0.97-1.24)	1.17 (1.03-1.32)	1.16 (1.03-1.31)
	Severe	1.23 (1.09-1.38)	1.25 (1.11-1.41)	1.34 (1.18-1.51)	1.34 (1.18-1.51)
Denture wearing, No					
	Stable	1 (Ref.)	1 (Ref.)	1 (Ref.)	1 (Ref.)
	Mild	0.97 (0.88-1.08)	0.97 (0.87-1.08)	0.99 (0.89-1.10)	0.98 (0.88-1.09)
	Middle	1.14 (1.02-1.28)	1.14 (1.02-1.28)	1.19 (1.05-1.34)	1.19 (1.06-1.35)
	Severe	1.13 (1.03-1.25)	1.16 (1.06-1.28)	1.25 (1.14-1.38)	1.35 (1.22-1.50)
Denture wearing, Yes					
	Stable	1 (Ref.)	1 (Ref.)	1 (Ref.)	1 (Ref.)
	Mild	0.79 (0.63-0.99)	0.80 (0.63-1.00)	0.80 (0.63-1.02)	0.80 (0.63-1.02)
	Middle	1.00 (0.78-1.27)	1.00 (0.79-1.28)	1.11 (0.87-1.41)	1.11 (0.87-1.41)
	Severe	0.96 (0.77-1.20)	0.97 (0.77-1.21)	1.02 (0.81-1.28)	1.04 (0.82-1.30)

Model 1: adjusted for age, sex, and enrollment year; Model 2: model 1 + further adjusted for province, residence, ethic, marriage status, occupation, education; Model 3: model 2 + further adjusted for ADL score, physical performance score, food diversity score, social activity score, and chronic disease score; Model 4: model 3 + further adjusted for number of teeth and denture wearing at baseline.

However, when stratified by denture wearing at baseline, the positive association of higher rate of tooth loss with risk of MCI was only observed among denture nonwearers (OR _middle vs stable_ = 1.19; 1.06-1.35; OR _severe vs stable_ = 1.35; 1.22-1.50). No association was observed among denture wearers (Table 3 and Figure 3).

## DISCUSSION

In this longitudinal study among elderly adults in China, we found that higher tooth loss rate was associated with an increased risk of MCI in a non-linear relationship, and severe rate of tooth loss was related to a 28% increased risk of MCI. The results were robust across a series of subgroup analyses by sex, age and number of teeth at baseline. However, the positive association of higher rate of tooth loss with MCI risk was disappeared among subjects with denture wearing at baseline. These findings added new evidence to the relationship between oral health and cognitive function.

Our results are consistent with previous studies in elderly adults. Previous longitudinal studies indicated that number of teeth at baseline was associated with cognitive decline among older adults [13, 14, 19]. Participants with more teeth suggested a slower pace of cognitive decline over time, when comparing with those with fewer teeth [14, 20]. However, another US longitudinal study reported that fewer teeth at baseline was not significantly associated with cognitive decline [17]. This inconsistency might be caused by difference in ethnicity, enrolled age distribution, follow-up period, and definition of cognitive decline. For example, the follow-up period to evaluate cognitive decline in the US cohort was only 2 years, [17] while the present study had a relatively longer follow-up period (median follow-up time: 5.93 years). Moreover, instead of using the teeth number at baseline as the exposure, our study used tooth loss rate as the exposure, which could capture the information on the dynamic change of the tooth with aging. In addition, the present study found that 19.34% participants wore denture at baseline, and that increased risk of MCI was disappeared among denture wearers, which suggested that denture wearing may provide an effect against the developing of MCI. Previous cohort studies also found that denture wearing provided a protective effect against death for all degrees of tooth loss. [16].

There are several possible mechanisms to explain the association between tooth loss and risk of MCI. First, periodontitis is a chronic inflammatory oral disease and a major cause of tooth loss [21]. It shares two pathological features of Alzheimer's disease namely oxidative damage and inflammation [22]. Oral pathogens and their toxic molecules, after disseminating into the bloodstream, may induce a low-grade systemic inflammation through upregulating the release of cytokines and inflammatory mediators, which can trigger neuroinflammation and cause neuronal degeneration [23–25]. Second, tooth loss may lead to poor nutrition status, caused by insufficient consumption of recommended levels of foods and nutrients. Poor nutrition status, such as vitamin B deficiency, has been suggested to be related to cognitive decline [26, 27]. Of note, denture wearing could effectively increase nutritional intakes, which may subsequently improve the cognitive function [28, 29]. Additionally, previous studies have shown that denture wearing could encourage social participation, which may in turn improve the cognitive performance [30]. Third, tooth loss may lead to decreased masticatory function. Mastication plays a key role in sending sensory information to the brain and in maintaining learning and memory functions of the hippocampus [31]. Masticatory dysfunction has been suggested to be associated with the hippocampal morphological impairments and cognitive decline in elderly adults [31, 32].

The strengths of the study included large sample size and abundant covariates, which enables us to construct different models and to conduct a series of subgroup analysis to test the robustness of the results. Meanwhile, teeth number and cognitive function were measured in each wave, so we could observe the trajectory of tooth changes and alterations of cognitive function during follow-up. Furthermore, our study was the first one to explore the dose-response association between number of tooth loss (per year) and risk of MCI. However, there are also several limitations. Although the Mini-Mental State Examination (MMSE) is the most widely used tool for identifying cognitive impairment, [33] its assessment may be biased due to differences of age, race, and education background [33]. Besides, there has a ceiling effect of MMSE, which may not be sensitive for MCI [33]. Second, self-reported teeth number may lead to misclassification of exposure status. However, self-reported teeth number has been widely used in previous epidemiological studies and strongly correlated with clinical records [34]. Third, lacking information on the detailed oral health, such as periodontal disease and denture type, makes us unable to explore whether the association between number of tooth loss and MCI was affected by those factors. Moreover, there was no detailed information on denture wearing during the follow-up, which may lead to misclassification of denture wearing to some extent as some participants may switch to other groups (e.g., from denture nonwearer to wearer). Therefore, replication from future study is needed, especially those with rich information on oral health. Fourth, individuals with MCI may be less likely to pay attention to their oral hygiene compared with those without MCI; reverse causation cannot be ruled out. Our findings therefore are only suggestive and cannot prove causality. Fifth, since this study was conducted in elderly adults in China, generalization of the findings to other population should be cautious.

In the present longitudinal study, we found that higher rate of tooth loss was associated with increased risk of MCI in elderly adults in China. Denture wearing however may provide a protective effect against the developing of MCI. Further studies are needed to investigate the potential mechanisms underlying the observed association. Our findings may have clinical implications on improvement of oral health to reduce potential risk of cognitive impairment in elderly adults.

## MATERIALS AND METHODS

### Study population

The Chinese Longitudinal Healthy Longevity Survey (CLHLS) is an ongoing longitudinal study of the elderly adults [35]. It started in 1998, and a series of follow-up surveys was conducted in 2000, 2002, 2005, 2008-2009, 2011-2012, 2014, and 2018-2019, respectively. In order to well-represent elderly adults in China and maintain enough sample size, new participants were added to the survey in the follow-up waves. We enrolled all participants (1998 wave), survivors and new participants (consequent waves) in the present study (n=56,570), and over 74% participants were firstly interviewed before 2011-2012 wave (Figure 1). Participants with the following reasons were excluded from the analysis: (1) immediately lost to follow up after baseline survey (n = 35); (2) birthday or end of follow up time missing (n = 94); (3) baseline age less than 65 years (n = 320); (4) education missing (n = 483); (5) no teeth at baseline (n = 6,955); (6) unable to assess change in teeth loss (n = 33,147); (7) having dementia at baseline (n = 752); (8) MCI missing (n = 627); or (9) having MCI at baseline (n = 2,295). Eventually, a total of 11,862 participants were enrolled in the final analysis. Among them, 6,886, 3,617, 1,608, 526, 29 and 5 participants had two, three, four, five, six and seven times of interview (Figure 1). Research Ethics Committees of Peking University approved the CLHLS, and written informed consents from all participants or their representatives were collected.

### Exposure, outcome and important covariates assessments

Self-reported information on number of natural teeth as well as the denture wearing (yes/no) was repeatedly collected and updated in each wave with the following questions: 1) How many natural teeth do you still have? 2) Do you have false teeth? Tooth loss rate was defined as the difference of teeth between two interview waves divided by years of interval. Participants were grouped into four categories by the following criteria: stable, no tooth loss; mild, 0-1 tooth loss; middle, 1-2 tooth loss; and severe, more than 2 tooth loss per year. The interviewer would help participants with hearing impairment to confirm their answers, which could largely ensure the validity of self-reported information on natural teeth and denture wearing.

Cognitive function was assessed by the Chinese version of MMSE at each wave. It contains twenty-four items regarding orientation, registration, attention, calculation, recall and language, with a total score ranging from 0 to 30; a higher score indicates a better cognitive function [36]. As cognitive performance was closely associated with educational level, different cut-off points across education levels were applied to define MCI: illiterate: < 18; those with level of primary school: < 21; and those with level of middle school or above: < 25 [37].

Age, gender and ethnicity were measured at baseline; all other covariates, such as residence, marriage status, occupation, education, smoking and drinking, activities of daily life (ADL) score, physical performance score, food diversity score, social activity score, and chronic disease score, were measured at baseline and at each follow-up interview. The definition of the abovementioned scores were described in previous study [38].

### Statistical analysis

Continuous variables were compared via ANOVA test, while categorical variables were compared via Chi-squared test. As one participant may have multiple records in the whole survey period and those records were statistically dependent, a generalized estimating equation (GEE) model was used to estimate the odds ratio (OR) and the 95% confidence intervals (CIs) with adjustment for potential confounders. In model 1, we adjusted for age, sex, and enrollment year. In model 2, we further adjusted for province, residence, ethic, marriage status, occupation and education. Covariates in model 2 plus the following covariates were further adjusted for in the model 3: ADL score, physical performance score, food diversity score, social activity score, and chronic disease score. Number of teeth at baseline and denture wearing were further adjusted for in the model 4. Subgroup analyses stratified by sex, age, number of teeth and denture wearing at baseline were also conducted to explore whether the results were consistent across different subgroup population.

To further explore the dose-response association between number of tooth loss per year and risk of MCI in the whole and subgroup population, restricted cubic spline function with 3 knots selected at number of tooth loss per year of “1, 2, 3” was applied in the GEE model. The reference value for number of tooth loss per year was zero.

All analyses were conducted using SAS 9.4 (SAS Institute Inc, Cary, NC, USA) and R platform. A *P* value less than 0.05 was considered statistically significant.
